# In contact with grief: Affectionate touch and intimacy in bereaved parents

**DOI:** 10.1016/j.ijchp.2024.100534

**Published:** 2024-12-12

**Authors:** Turan Deniz Ergun, Asuman Buyukcan-Tetik, Anik Debrot, Henk Schut, Margaret Stroebe

**Affiliations:** a Department of Psychology, Health, & Technology, University of Twente, The Netherlands; b Psychology Program, Sabanci University, Turkey; c Department of Clinical Psychology, Utrecht University, The Netherlands; d Institute of Psychology, University of Lausanne, Switzerland; e Department of Clinical Psychology and Experimental Psychopathology, University of Groningen, The Netherlands

**Keywords:** Affectionate touch, Intimacy, Child loss, Diary, Dyadic design

## Abstract

Although child loss impairs well-being, its impact on behavioral exchanges between bereaved parents remains understudied. We compared bereaved and non-bereaved couples regarding affectionate touch levels, the role of affectionate touch in intimacy, and the association between partners’ affectionate touch similarity and intimacy. Bereaved (228 couples, 27 individuals) and non-bereaved (258 couples, seven individuals) people participated in our seven-day diary study. Although bereaved and non-bereaved men reported equal affectionate touch, bereaved women's affectionate touch was lower than non-bereaved women's. Despite this discrepancy, multilevel analyses revealed that affectionate touch concurrently benefited both genders’ intimacy in bereaved and non-bereaved couples. For bereaved women, touch also contributed to next day's intimacy. We also showed that couples reported higher intimacy if both partners had higher vs. lower affectionate touch. Our findings highlight bereaved and non-bereaved couples’ similarity regarding the relational gains of affectionate touch and the promising function of affectionate touch in coping with loss.

## Introduction

Child loss is one of the most painful and traumatic events an individual can experience throughout life (Fernández-Alcántara, 2017; Rogers et al., 2008). It can occur during pregnancy, labor, or when the child is older (e.g., infant, adult). After child loss, parents’ worldview about the expected natural progress of events regarding having and raising a child may be disrupted, and their understanding of predictability and security may fall apart (Rubin & Malkinson, 2001).

Such an experience also brings detrimental health consequences, regardless of its timing (Goldstein et al., 2018; Pohlkamp et al., 2019). For example, bereaved parents report lower levels of well-being (Rogers et al., 2008) and poorer health-related life quality than their non-bereaved counterparts (Song et al., 2010), also after a considerably long time since the loss (e.g., Horesh et al., 2018; Rubin, 1993). In this study, we focus on the experience of child loss at any stage of the child's life, following similar applications in bereavement research (e.g., Dyregrov & Dyregrov, 2017).

The loss of a child also threatens couples' relationships. For example, previous studies have highlighted the association of child loss with higher marital distress (see Albuquerque et al., 2016, for a review). Nevertheless, some protective factors may buffer the detrimental effects of losing a child on bereaved parents' individual and relationship well-being. For example, parents' involvement in dyadic coping strategies, such as supportive stress communication, positively relates to bereavement adjustment (Bergstraesser et al., 2015). However, especially under trying circumstances, providing verbal support can be challenging for the partners (Jakubiak, 2021).

In this research, we aim to investigate whether another type of supportive behavior, namely affectionate touch, could promote bereaved parents' relationship quality. Affectionate touch is defined as touching behaviors aiming to provide love and affection to the receiver, such as hugging and kissing (Jakubiak & Feeney, 2017), which may be enacted with sexual and/or non-sexual intentions. Previous correlational and some causal studies have emphasized the beneficial role of affectionate touch in psychological (e.g., lower negative mood; Burleson et al., 2007), physiological (e.g., cortisol recovery; Ditzen et al., 2019; see Floyd et al., 2020 for a review), and relational well-being (e.g., Carmichael et al., 2021; Durbin et al., 2021).Nevertheless, past studies have not considered real-life stressful circumstances (e.g., major life events) that may impact affectionate touch behaviors. This lack of research is surprising because such contexts shape the enactment and evaluation of behavioral exchanges between partners (e.g., Hobfoll & Spielberger, 1992; Neff & Karney, 2017), such as increasing criticizing and blaming behaviors (Neff & Buck, 2023).

In the current study, we aimed to extend research on affectionate touch to couples who experienced child loss. Support-seeking and -provision behaviors could be vital in such a context to reconstruct the feelings of security damaged due to being permanently detached from such a strong bond (Mikulincer & Shaver, 2009; Sbarra & Hazan, 2008). Indeed, affectionate touch, which is a reciprocal act by its nature, was shown to serve as a non-verbal behavior promoting safety in partners (Jakubiak & Feeney, 2016, 2017; Murray, 2023). This non-verbal behavior is preferred over verbal behaviors by both support providers and recipients (Jakubiak, 2021; Jakubiak & Feeney, 2016). Considering these pieces of evidence, affectionate touch is a behavior that deserves close attention in the context of child loss.

### Affectionate touch in bereaved vs. non-bereaved couples

The first aim of this study was to compare bereaved and non-bereaved couples regarding their affectionate touch levels. Previous research has revealed a negative association between traumatic events and physical and emotional intimacy (Mills & Turnbull, 2004; Riggs, 2014; Whisman, 2006). However, the link between affectionate touch and intimacy was never tested in bereaved people. Studies conducted on sexuality after child loss have revealed that the sexual intimacy of bereaved parents decreases over time (Gottlieb et al.,1996). Similarly, in a qualitative study with twenty-four bereaved parents, the majority reported that the frequency of sexual intercourse declined after their child loss (Hagemeister & Rosenblatt, 1997). Although affectionate touch is not necessarily enacted for sexual purposes, these results reveal the reduced physical intimacy between bereaved parents after their child's death. Considering these findings, we expected lower affectionate touch behaviors in bereaved couples than in non-bereaved couples.

### Affectionate touch and intimacy in bereaved vs. non-bereaved couples

The second aim of this research was to examine whether affectionate touch was similarly associated with intimacy in bereaved vs. non-bereaved heterosexual couples. Although the link between affectionate touch and intimacy is presumably bidirectional, Jakubiak and Feeney (2017) proposed that receiving affectionate touch specifically contributes to relational, psychological, and physical well-being and buffers stress reactivity through several pathways. As a neurobiological pathway, affectionate touch stimulates neurological and biological changes that enhance well-being and lead to reductions in stress. As a relational-cognitive pathway, affectionate touch reinforces the receiver's intention to feel close to and trust the partner. Indeed, affectionate touch behaviors and partners' perceived responsiveness are mutually influential (Jolink et al., 2021), and affectionate touch predicts both provider's and receiver's intimacy in daily diary and experimental studies (Debrot et al., 2013; Durbin et al., 2021).

Could affectionate touch similarly enhance intimacy in bereaved parents? We propose that affectionate touch may matter for bereaved parents, who could potentially experience feelings of anxiety, uncertainty, insecurity, and loneliness (Campbell-Jackson et al., 2014; Essakow & Miller, 2013; Murphy et al., 2014; Rosenberg et al., 2012; Wang & Hu, 2022) and serve as a nonverbal support behavior. Bereaved parents may benefit from reduced stress reactivity and pain, felt security and comfort, and heightened intimacy thanks to receiving touch from their partner (Debrot et al., 2013; Durbin et al.,2021; Goldstein et al., 2017; Grewen et al., 2003; Sahi et al., 2021). Several experimental studies have shown the soothing role of touch. For instance, Robinson et al. (2015) showed that touch is a nonverbal instrument of communication used to solicit and provide a feeling of support between partners. These findings indicate that affectionate touch can be comforting in distressed couples' daily lives, which we aim to examine by testing the link between affectionate touch and intimacy for bereaved couples in this research. However, we did not have any evidence to propose a significant difference between the bereaved and non-bereaved couples regarding the strength of this association.

### Partners’ similarity in affectionate touch

As the third aim of this research, we investigated if the association between affectionate touch and intimacy depends on both partners engaging in similar levels of affectionate touch. What happens to the intimacy level when the couples' affectionate touch behaviors are not at equal levels? On the one hand, a little affectionate touch from the receiver in response to high levels of affectionate touch from the provider could lead to disappointments and harm intimacy on the provider's side (cf. Gleason et al., 2003; Sprecher et al., 1986). On the other hand, the positive impact of touch seems to occur even when people are unaware that they were touched (Fisher et al.,1976; Robinson et al., 2015). Perhaps touch positively impacts intimacy even when one partner's touch level is not equal to the other partner's. Thus, we will explore the association of the similarity of affectionate touch across partners with intimacy.

### Gender and culture

Although both bereaved parents go through a traumatic experience, interestingly, research primarily focused on women and has not paid enough attention to men (Lamon et al., 2022; Mcneil et al., 2021). Bereaved women are mainly considered to be the support receivers from their partners according to societal norms (Cimete & Kuguoglu, 2006; Dyregrov, 1990; Tanacıoğlu-Aydın & Erdur-Baker, 2022). Bereaved men, however, are generally expected to stay strong, which may lead them to suppress their emotions and avoid grief-related discussions with their partner (Cook, 1988; Dyregrov & Matthiesen, 1991; Dyregrov et al., 2020; Mandell et al., 1980). In such a context, parents likely engage in affectionate touch behaviors to provide nonverbal support to or get support from their partner. Because of its reciprocal nature in romantic relationships, frequent affectionate touch could be a tool to compensate for the disrupted communication between bereaved parents (Albuquerque et al., 2018). Therefore, in the present study, we considered a dyadic approach and investigated the association of one partner's affectionate touch with their and their partner's intimacy.

Another limitation of bereavement literature is that most studies have been conducted in Western countries, although bereaved parents' grief responses may vary across cultures (Mcneil et al., 2020; Stroebe & Schut, 1998; Zhou et al., 2023). Filling that gap, we conducted our study in a non-Western and predominantly Islamic context: Turkey (Nişancı et al., 2023). Because a child is also a source of social status in Turkey, bereaved parents may experience particularly intense reactions in response to child bereavement (Cimete & Kuguoglu, 2006). Nevertheless, some parents are likely to avoid explicitly showing their grief because public grief expressions may be perceived as an act of disobedience against God's will in conservative groups (Rubin & Yasien-Ismael, 2004; Tanacıoğlu-Aydın & Erdur-Baker, 2022). As a result, affectionate touch may play a non-verbal supportive role in such a relatively conservative context.

### The present study

We examined three research questions in this study. We first sought to examine whether bereaved parents' (i.e., Loss Group) average affectionate touch levels are similar to non-bereaved participants’ (i.e., Comparison Group) affectionate touch levels and expected that bereaved women and men report lower levels of average affectionate touch compared to non-bereaved women and men, respectively. Second, we aimed to examine the actor effects (i.e., whether one's affectionate touch is associated with their own intimacy) and partner effects (i.e., whether one's affectionate touch is related to their partner's intimacy). We hypothesized that affectionate touch positively relates to both partners’ intimacy in both Loss and Comparison Groups. Lastly, using a couple-level approach, we explored whether the benefits of affectionate touch for intimacy depend on affectionate touch similarity between partners (i.e., both partners engaging in highly affectionate touch) in both bereaved and non-bereaved couples. We examined these questions through a seven-day dyadic diary conducted in Turkey, an underrepresented cultural context. We preregistered our hypotheses and data analytic strategy (https://osf.io/m93vj/). The results of the non-preregistered exploratory analyses are presented in a separate section.

## Method

### Procedure and participants

We collected data from married heterosexual couples who experienced child loss during pregnancy, labor, or afterward (i.e., Loss Group) and those who did not experience child loss (i.e., Comparison Group) between August 2020 and December 2021 as part of a larger project (Buyukcan-Tetik et al., 2023). The Research Ethics Council of the first author's former institution approved the research. We recruited participants by multiple methods, including contacting the research assistants' social networks, posting online announcements and advertisements about our project on different social media platforms, forums about child loss, and on our lab's website, and communicating with clinicians and medical doctors. The announcements included a link to our lab's website, where potential participants could fill in an application form, which included a consent form and questions about partners' names, email addresses, and phone numbers. Later, our research team phoned participants who completed the application form to verify their availability and willingness to participate in the study as a couple. We asked participants to think of their most recent loss in case of multiple losses.

Our data cleaning procedure consisted of multiple steps (e.g., participants who gave wrong responses to attention-check questions were excluded; see the Supplemental Materials for details) to identify and exclude participants accordingly. After the data cleaning steps, the Loss and Comparison Groups consisted of 228 couples and 27 individuals (*N* = 483) and 258 couples and seven individuals (*N* = 523), respectively. Please note that the final dataset also included some individual participants because their partners did not complete the surveys. Most of the families experienced pregnancy loss (70%), whereas the remaining families experienced child loss during the labor or days, months, or years afterward. Moreover, our sample mostly included participants who are middle-aged and relatively highly educated and identified themselves as moderately religious, albeit with some differences across Loss and Comparison Groups. Detailed sample characteristics are presented in Table 1. We also provided the descriptive statistics of individual and relational well-being variables, as well as the frequencies for religiosity and socioeconomic status levels, in our cross-sectional survey, in the Supplemental Materials to promote understanding of the sample characteristics. From now on, throughout the text, we consider bereaved mothers and fathers as bereaved women and men to ensure consistent use of participants’ gender across Loss and Comparison Groups.

**Table 1:** Sample Characteristics.

	Loss Group			Comparison Group		
	*M*	*SD*	*Range*	*M*	*SD*	*Range*
Age (W)	40.43	10.48	20–74	33.83	8.27	22–59
Age (M)	44.48	11.05	25–57	36.08	8.56	23–65
Marriage length (years)	16.63	11.36	.67–56	8.14	8.73	.08–40.58
Number of living children (W)	1.48	1.20	0–10	0.93	0.96	0–4
Number of living children (M)	1.45	1.24	0–10	0.95	0.97	0–4
Number of lost children (W)	1.49	0.97	1–8	–	–	–
Number of lost children (M)	1.45	0.91	1–6	–	–	–
Time passed since the loss (years)	10.77	10.01	.08–50	–	–	–
Deceased child's age (years)	3.29	6.58	0–25	–	–	–
Pregnancy month	3.28	1.69	.75–9	–	–	–
Education (W)	4.58	1.60	1–7	5.87	1.22	2–7
Education (M)	4.73	1.51	2–7	5.71	1.20	2–7
Socioeconomic status (W)	5.45	1.87	1–10	5.83	1.49	1–9
Socioeconomic status (M)	5.43	1.74	1–9	5.64	1.64	1–10
Religiosity (W)	4.74	1.52	1–7	4.05	1.65	1–7
Religiosity (M)	4.59	1.68	1–7	4.02	1.83	1–7

*Note. W* = Women, *M* = Men. The number of living children slightly differed across women and men, possibly because some participants had children from previous marriages. The deceased child's age indicates the age of the child who died during labor or afterward. The pregnancy month variable represents the gestational period for the pregnancy losses. The number of lost children includes experienced pregnancy and child losses. We measured socioeconomic status by asking participants to rate their socioeconomic status on a 10-step ladder (Adler & Stewart, 2007). Education was assessed using a 7-point question representing increasing levels of education (1 = Literate but no formal education, 7 = master's or Ph.D. degree). Religiosity was examined by asking a single 7-point question on to what extent the participants identified themselves as religious (1 = Not at all, 7 = Very much).

The data collection through Qualtrics had two stages: a cross-sectional survey and a 7-day diary. We asked the participants to start the diary survey a week after they completed the cross-sectional survey. Depending on participants' availability, the average number of days between these two stages was 8.18 (*SD* = 3.84) and 7.88 (*SD* = 2.23) for the Loss and Comparison Groups, respectively. We emailed the daily surveys’ links to the participants at 7:00PM and asked them to complete them by midnight. Each participant was compensated with a shopping voucher for up to 100 Turkish Liras (USD 13.72 by August 2020), depending on their survey completion rates. The average number of completed surveys was 5.80 for the Loss Group and 5.97 for the Comparison Group. We report all exclusions in the Supplemental Materials.

### Measures

We measured daily affectionate touch and intimacy using single items as applied in previous diary studies in relationship science (e.g., Debrot et al., 2012; Laurenceau et al., 2005; Le et al., 2019). The item “Today, I touched my partner in an affectionate and caressing manner (e.g., hugged)" assessed affectionate touch (Debrot et al. 2012), and the item "Today, I felt close to my partner" measured intimacy. Participants answered both questions on a 5-point Likert scale (1 = *Strongly disagree,* 5 = *Strongly agree*). For all variables, including the questions related to loss-related characteristics and grief symptoms, measured in our surveys, please see the OSF page (https://osf.io/m93vj/).

### Strategy of analysis

We conducted all our analyses using Mplus 8.4 (Muthén & Muthén, 1998-2019) and the full information maximum likelihood estimation with robust standard errors (i.e., MLR) to handle missing data (Enders & Bandalos, 2001). This allowed us to use the full available data, even if one partner was missing in some couples (Young & Johnson, 2013). Below, we report the analysis strategy for each research question separately.

#### Research question 1

For the first research question (RQ1), we conducted Wald chi-square tests to compare the average affectionate touch levels across seven days of the diary between Loss and Comparison Groups. Thereby, we examined the within-gender differences between the groups (i.e., affectionate touch of women in the Loss Group vs. *women* in the Comparison Group). We did not compare across-gender differences between the groups (i.e., affectionate touch of women in the Loss Group vs. *men* in the Comparison Group). Within-group gender differences were detected in the scope of the descriptive statistics (i.e., affectionate touch of women vs. men in the Loss Group).

#### Research question 2

**Fig. 1:**
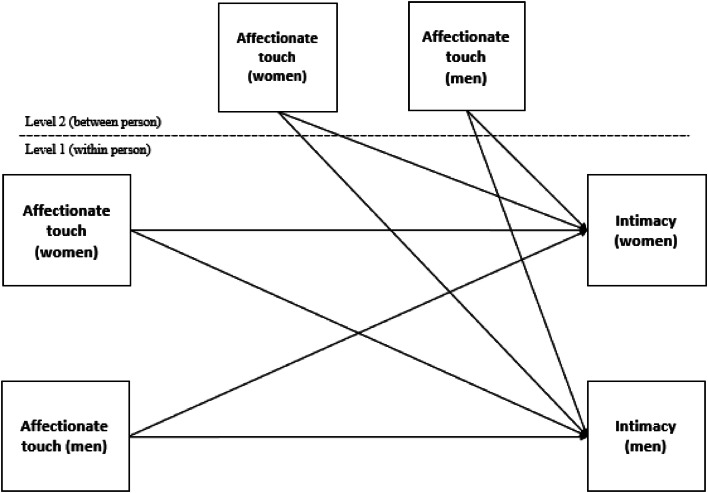
The Conceptual Multilevel Actor-Partner Interdependence Model.

*Note*. In our multilevel Actor-Partner Interdependence Model, we included both the person-mean centered within-level and grand-mean centered between-level affectionate touch variables.

For the second research question (RQ2), we conducted a multilevel Actor-Partner Interdependence Model (APIM; Kenny et al., 2020) analysis to test the association between daily affectionate touch and intimacy by considering the nested nature of the data (daily observation within individuals and individuals within couples). We included person-mean centered levels at each day as the Level 1 within-person variables and grand-mean centered averages across the diary as the Level 2 between-person variables in our multilevel models (Bolger & Laurenceau, 2013; Nezlek, 2007). These within- and between-person variables reflected the state and trait-like levels, respectively. Because we benefited from a multilevel APIM framework, we regressed the outcome variable (i.e., intimacy) on both partners’ within- and between-person affectionate touch variables (see Fig. 1). Moreover, we estimated random slopes for the actor and partner effects and hence allowed those effects to vary across participants. We conducted a chi-square difference test to examine whether the effects could be constrained to be equal across Loss and Comparison Groups in the same multilevel model. A significant chi-square difference meant that the effects should not be constrained across groups and should be freely estimated. In that case, we again kept the analyses for both groups within the same multilevel model, and both groups were allowed to produce different estimates. Although our research question focused on the concurrent association between affectionate touch and intimacy on the same day, we also explored the lagged effects of one day's affectionate touch (intimacy) on the following day's intimacy (affectionate touch) in subsequent analyses to detect the direction of the relation.

We first investigated the variance of each variable explained at the within- and between-levels. In the Loss Group, slightly higher than half of the variance for affectionate touch (55% for women, 51% for men) and intimacy (52% for women and 56% for men) depended on within-person variations. Similarly, in the Comparison Group, the variance of affectionate touch (58% for women, 62% for men) and intimacy (60% for women, 59% for men) was predominantly due to the changes at the within-person level. We considered the interdependence between women's and men's within- and between-person variables in the same model and investigated the relation of participants’ affectionate touch with their (i.e., actor effect) and their partner's (i.e., partner effect) intimacy.

#### Research question 3

**Fig. 2:**
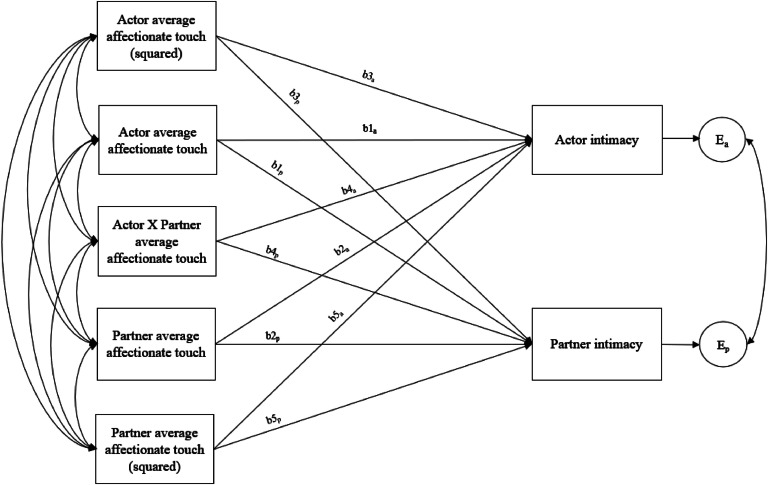
The Dyadic Response Surface Analysis Conceptual Model.

*Note. a* = Actor, b1-b5 = Regression coefficients, *e* = Error term, *p* = Partner. All affectionate touch variables are grand mean centered. As elaborated in the manuscript, there were no significant differences in b1-b5 coefficients across women and men, and hence, we present the variables as actor and partner variables in this figure.

To test whether the association between affectionate touch and intimacy depended on both partners' engagement in similar levels of affectionate touch for our third research question (RQ3), we conducted a Dyadic Response Surface Analysis (DRSA; Schönbrödt et al., 2018). In the DRSA, we regressed women's and men's average intimacy on women's and men's average affectionate touch across the diary (i.e., linear effects), their squared terms, and the interaction between linear effects (Fig. 2). Before running the DRSA, we confirmed that there was variance in the data regarding the difference between partners' affectionate touch levels (Shanock et al., 2010; see Supplemental Materials). We grand-mean centered the affectionate touch variable (Schönbrödt et al., 2018).

Regression coefficients (b1-b5; Fig. 2) in the DRSA are used to compute five parameters (a1-a5). The parameters a1 and a2 represented the linear and non-linear effects for the *line of congruence* (i.e., the line where partners' affectionate touch levels are equal, e.g., both partners' affectionate touch scores were 1 unit higher than the grand-mean), respectively. The parameters a3 and a4 reflected the same effects for the *line of incongruence* (i.e., the line where the partners equally deviate from the grand-mean but in opposite directions, e.g., one partner's affectionate touch score was 1 unit lower than the grand-mean vs. the other partner's affectionate touch score was 1 unit higher than the grand-mean). Lastly, parameter a5 indicates how the ridge line of the response surface is located compared to the line of congruence.

To test whether the similarity of affectionate touch between partners is associated with higher levels of intimacy, the following criteria are detected: *a1 > 0, a2 = ns, a3 = ns, a4 < 0, a5 = ns* (Humberg et al., 2019; Schönbrödt et al., 2018)*.* A positive a1 suggests that participants report higher levels of intimacy than other participants in the sample when both partners of a couple have similarly higher (vs. similarly lower) average affectionate touch levels. A non-significant a2 indicates that partners' similar levels of affectionate touch at extremes (e.g., both partners’ affectionate touch scores are 1) vs. the grand-mean have the same effect on intimacy. A non-significant a3 suggests that participants' intimacy is not affected by whether they have higher levels of affectionate touch than their partner or their partner has higher affectionate touch levels than themselves. A negative a4 indicates that partners who had differences at the extremes (e.g., one partner's affectionate touch score of 5 vs. the other partner's affectionate touch score of 1) had lower intimacy than partners both of whom had the grand-mean. Lastly, a5 must be nonsignificant if the similarity criteria of a1-a4 are met, representing that the ridge of the surface is in the same position as the line of congruence.

### Power

We first conducted a power analysis for the Actor-Partner Interdependence Model using APIMPowerR (Ackerman et al., 2016) using the effect sizes found in non-bereaved couples (Debrot et al., 2013). Our sample size provided a power higher than 0.80 for both Loss and Comparison Groups. In addition, we checked the multilevel power curves suggested by Bolger and Laurenceau (2013) and found that our sample size was sufficient to detect medium effect sizes with a power higher than 0.80 (see Supplemental Materials). Although no criterion for the sample size was set to run DRSA, at least 200 participants are needed for non-dyadic Response Surface Analysis (Schönbrödt et al., 2018). The sample sizes in the dyadic datasets of the Loss and Comparison Groups doubled that number.

## Results

### Descriptive statistics and correlations

Descriptive statistics of and correlations between study variables are presented in Table 2. The correlations between the study variables were positive and significant for both the Loss and Comparison Groups. Men reported significantly higher levels of affectionate touch than women on average (Loss Group: Wald = 27.17, *df* = 1, *p* ≤ .001, *d* = 0.33^1^; Comparison Group: Wald = 6.08, *df* = 1, *p* = .014, *d* = 0.13). Similarly, men reported higher levels of average intimacy compared to women in both the Loss (Wald = 37.33, *df* = 1, *p* ≤ .001, *d* = 0.40) and Comparison Groups (Wald = 17.23, *df* = 1, *p* ≤ .001, *d* = 0.25).

**Table 2:** Descriptive statistics of and correlations between study variables.

	*Loss Group*					*Comparison Group*
Variable	*M (SD)*	*1*	*2*	*3*	*4*	*M (SD)*
1. Affectionate touch (W)	3.68 (1.06)	–	.60	.71	.51	3.92 (1.02)
2. Affectionate touch (M)	4.01 (0.95)	.60	–	.46	.75	4.05 (0.89)
3. Intimacy (W)	3.98 (0.90)	.80	.47	–	.55	4.12 (0.82)
4. Intimacy (M)	4.31 (0.73)	.53	.77	.53	–	4.31 (0.71)

*Note.* Descriptives show the averages across seven days of the diary. The correlations below and above the diagonal line belong to the Loss and Comparison Groups, respectively. *W* = Women, *M* = Men. All correlations were significant at *p*<.001 level.

### RQ1: Comparison of affectionate touch across the groups

Wald chi-square tests revealed that women in the Loss Group reported significantly lower average affectionate touch than women in the Comparison Group (Wald = 6.34, *df* = 1, *p* = .012, *d* = 0.23; see Table 2 for the averages). Considering the difference in relationship length across groups (Table 1), we re-ran this analysis controlling for relationship length. In that analysis, the difference between women across groups became marginally significant (Wald = 3.185, *p* = .074). However, we did not find a significant difference in men's average affectionate touch between the Groups in the analyses without (Wald = 0.46, *df* = 1, *p* = .497) or with relationship length (Wald = 0.45, *df* = 1, *p* = .505).

### RQ2: The association between affectionate touch and intimacy

The results of the multilevel models are presented in Table 3. Before conducting the analysis, we used the chi-square difference test to examine whether the between- and within-person effects can be constrained to be equal across the Loss and Comparison Groups in the same model. The chi-square difference test revealed that unconstrained and constrained models were significantly different from each other (Δχ²(22, *N* = 1006) = 103.77, *p* < .001). Consequently, we included both Loss and Comparison Groups in the same model but conducted a multigroup analysis. A multigroup analysis gives identical results to an interaction analysis (Curran, 2003) with a moderating group variable (i.e., loss group vs. comparison group). To enhance readability, we reported the results for Loss and Comparison Groups in separate columns in tables.

**Table 3:** Multilevel model results for affectionate touch's effect on intimacy in Loss and Comparison Groups.

	Loss Group			Comparison Group		
	*b*	*p*	95% CI	*b*	*p*	95% CI
**Within-person level**						
Actor effect (W)	**.41**	**<.001**	**[.35 - .47]**	**.43**	**<.001**	**[.36 - .49]**
Actor effect (M)	**.40**	**<.001**	**[.33 - .46]**	**.37**	**<.001**	**[.31 - .43]**
Partner effect (W)	**.06**	**.004**	**[.02 - .10]**	**.09**	**<.001**	**[.05 - .14]**
Partner effect (M)	.05	.091	[−.01 - .12]	**.09**	**.002**	**[.03 - .14]**
**Between-person level**						
Actor effect (W)	**.70**	**<0.001**	**[.60 - .81]**	**.58**	**<.001**	**[.47 - .69]**
Actor effect (M)	**.51**	**<0.001**	**[.38 - .63]**	**.56**	**<.001**	**[.44 - .69]**
Partner effect (W)	**.09**	**.014**	**[.02 - .17]**	.04	.343	[−.05 - .13]
Partner effect (M)	−.02	.706	[−.13 - .09]	.02	.809	[−.11 - .14]

*Note. W* = Women, *M* = Men. The bold results are significant. We included the Loss and Comparison Groups in the same model and conducted multigroup analysis to produce the estimates for each group.

For the Loss Group, at the within-person level, both women and their partners had a higher level of intimacy on days when women's affectionate touch level was higher than their average across days, compared to the days when women's affectionate touch was lower than their average. The between-person level results indicated that women with higher affectionate touch on average reported higher intimacy on average and were also married to men with higher intimacy on average. For men, only the actor effects were significant at the within-person level, revealing that men had higher intimacy when their affectionate touch was higher (vs. lower) than their average. Nevertheless, men's deviation from their average affectionate touch was not associated with their partner's intimacy. Similarly, at the between-person level, compared to men with lower average affectionate touch, men with higher average affectionate touch reported higher intimacy, but their partner did not. Moreover, we found significant variances in the random slopes for men's (σ^2^ = 0.07, *p*< .001) and women's (σ^2^ = 0.08, *p*< .001) actor effects, indicating heterogeneity in the strength of the relationship between bereaved parents’ affectionate touch and their intimacy. Partner effects did not have a significant variance, showing the existence of a similar association between affectionate touch and the partner's intimacy across participants.

For the Comparison Group, the actor and partner effects of women were again significant at the within-person level, suggesting that when women reported higher (vs. lower) affectionate touch than their average, they and their partner reported higher levels of intimacy. We found a significant actor effect (but no partner effect) at the between-person level: women with higher average affectionate touch reported higher intimacy than women with lower average affectionate touch. We also found significant actor and partner effects of men's affectionate touch at the within-person level: Men and their partner reported higher levels of intimacy on the days men engaged in higher (vs. lower) than their average affectionate touch. At the between-person level, however, only the actor effect was significant, showing that men with higher average affectionate touch reported higher intimacy than men with lower average affectionate touch.

Moreover, we also conducted the same multilevel analysis while excluding individual participants from the dataset. Removing individual participants did not prominently change the results either for the Loss or Comparison Group. For the input and output files of the analyses with and without individual participants, please see our OSF page (https://osf.io/m93vj/).

In further analysis, we tested whether within-person level associations between affectionate touch and intimacy in each group significantly differed across women and men. The results did not reveal any gender differences (Wald test *p*s = 0.199–0.998). Moreover, we conducted Wald chi-square difference tests for significant actor and partner effects in Table 3 to explore if they varied across Loss and Comparison Groups. For example, we compared women's within-person actor effects in the Loss vs. Comparison Groups. The results did not reveal any significant differences regarding the strengths of the effects across the groups (Wald test *p*s = 0.105–0.249).

Overall, at the within-person level, women's and men's actor and partner effects were positive for both the Loss and Comparison Groups, except for the non-significant partner effect of men in the Loss Group. However, the gender difference in partner effects should be interpreted cautiously, considering that the lower bound of the confidence interval is nearly zero for women's partner effect on men's intimacy, and Wald tests did not reveal a significant difference between women's significant and men's non-significant partner effects at the within-person level. At the between-person level, however, while women's and men's actor effects were positive for both groups, only women's partner effect was significant in the Loss Group. Please see Supplemental Materials for a summary of the results.

### RQ3: The role of affectionate touch similarity in intimacy

We first tested the equality of DRSA models of Loss and Comparison Groups (Fig. 2). The chi-square difference test revealed that the estimates for the Loss and Comparison Groups were significantly different and could not be constrained to be equal (Δχ²(22, *N* = 1006) = 514.76, *p* < .001). Thus, we estimated the effects for Loss and Comparison Groups in the same model but reported the results for each group in separate columns in tables. We also checked whether each within-group actor and partner effects differed across genders. Because Wald tests did not reveal a significant gender difference in either group (see Supplemental Materials), we constrained the actor and partner effects to be equal across women and men.

**Fig. 3.1:**
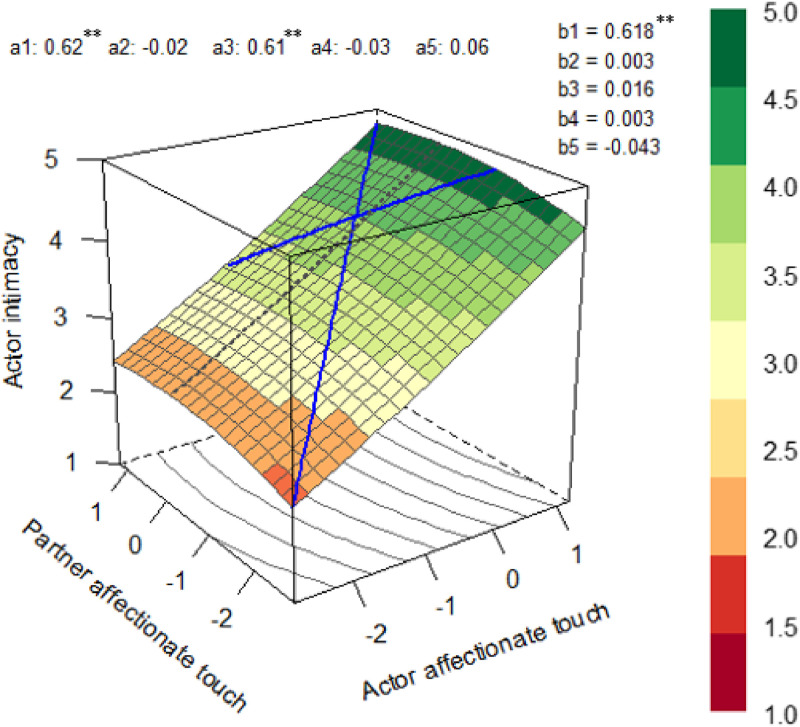
The Dyadic Response Surface Graph for the Loss Group.

*Note.* **p < .001. All affectionate touch variables were grand mean centered.

Table 4 presents the DRSA results. For both Loss and Comparison Groups, we did not find evidence indicating that the similarity of affectionate touch levels between partners was associated with higher levels of intimacy. However, we found positive a1 parameters for Loss and Comparison Groups, showing that the same affectionate touch level across partners was positively associated with intimacy. Our results also revealed positive a3 parameters for both groups, which showed that participants reported higher levels of intimacy, on average, than other participants when their affectionate touch was higher (vs. lower) than their partner's. Moreover, we found a significant a5 effect for the Comparison Group, indicating that the ridge of the surface graph was in a different position than the line of congruence. Please see Figs. 3.1. and 3.2. for the response surface graphs for the Loss and Comparison Groups, respectively.

**Table 4:** Dyadic Response Surface Analysis for the association between affectionate touch and intimacy.

	Loss Group			Comparison Group		
	*b*	*p*	95% CI	*b*	*p*	95% CI
**Polynomial regression coefficients**						
b1 actor	**.62**	**<.001**	**[.55 - .68]**	**.38**	**<.001**	**[.27 - .49]**
b2 partner	.00	.910	[−.06 - .06]	**.14**	**.009**	**[.03 - .24]**
b3 actor^2^	.02	.627	[−.05 - .08]	−.06	.228	[−.17 - .04]
b4 actor*partner	.00	.932	**[−.07 - .07]**	.01	.899	[−.15 - .18]
b5 partner^2^	−.04	.057	[−.09 - .00]	**.09**	**.023**	**[.01 - .17]**
**DRSA parameters**						
a1 (LOC, linear)	**.62**	**<.001**	**[.55 - .69]**	**.52**	**<.001**	**[.42 - .60]**
a2 (LOC, quadratic)	−.02	.593	[−.11 - .07]	.04	.568	[.−.09 - .16]
a3 (LOIC, linear)	**.61**	**<.001**	**[.51 - .72]**	**.25**	**.009**	**[.06 - .43]**
a4 (LOIC, quadratic)	−.03	.645	[−.16 - .10]	.02	.924	[−.29 - .31]
a5	.06	.085	[−.01 - .13]	**−0.15**	**<.001**	**[−.24 - −.07]**
**Evidence for the similarity? (a1 > 0, a2 = ns, a3 = ns, a4 < 0, a5 = ns)**						
	No, a3 = not ns, a4 = ns			No, a3 = not ns, a4 = ns, a5 = not ns		

*Note.* LOC = Line of congruence, LOIC = Line of incongruence, CI = Confidence interval, ns = non-significant. The bold results are significant. RSA parameters are calculated by the following formulas: a1 = b1+b2; a2 = b3+b4+b5; a3 = b1-b2; a4 = b3-b4+b5; a5 = b3-b5. We included the Loss and Comparison Groups in the same model and conducted a multigroup analysis to produce free estimates for each group.

**Fig. 3.2:**
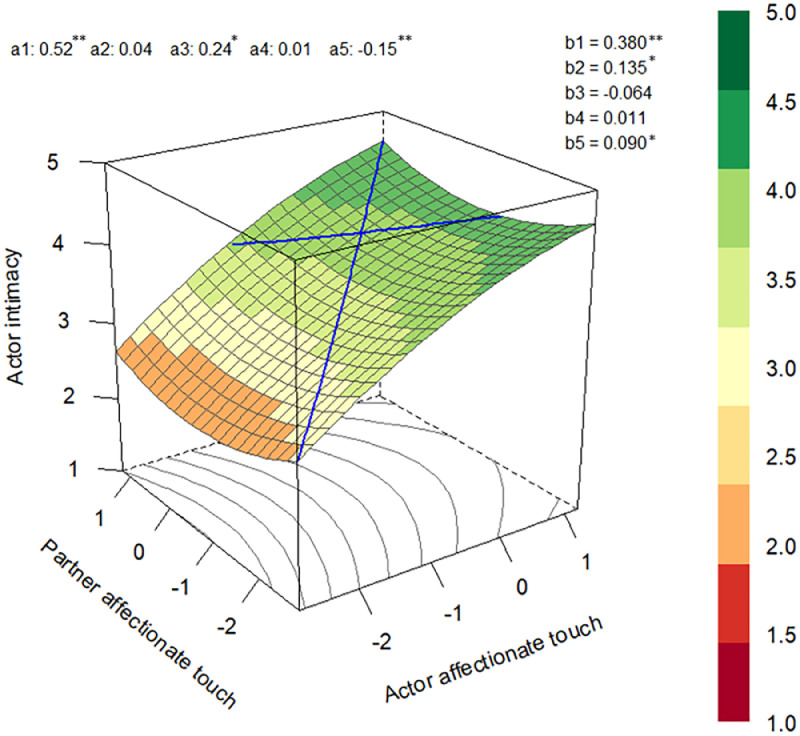
The Dyadic Response Surface Graph for the Comparison Group.

*Note.* **p < .001, *p < 05. All affectionate touch variables were grand mean centered. Both Fig. 3.1. and 3.2. were produced using the “RSA” R package (Schönbrödt & Humberg, 2023).

### Subsequent analyses

As another pre-registered examination, we tested whether the relevant control variables that may influence our results (participants' age, socioeconomic status, education level, number of living children, deceased child's age, pregnancy month of pregnancy loss, time since the loss, experience of one vs. more child losses) impacted the association between affectionate touch and intimacy. Because participants' age and time since loss were strongly correlated (*r*_women_ = 0.809, *r*_men_ = 0.795), we controlled for the effect of these variables in separate models. Adding these variables did not prominently change the results reported for the original models.

### Exploratory analyses

As exploratory analyses, we tested the lagged associations between affectionate touch and intimacy in separate models to understand the direction of the association (see Table 5). To do so, we regressed intimacy on the preceding day's affectionate touch and affectionate touch on the previous day's intimacy in separate models while keeping the multilevel structure of the data. Because we controlled for the effect of the outcome (e.g., intimacy) on the preceding day, the results showed whether the predictor (e.g., affectionate touch) was associated with a change in the outcome between two days. The models for either direction mostly revealed non-significant actor and partner effects at the within-person level for both men and women across Loss and Comparison Groups, but a positive actor effect of women's affectionate touch in the Loss Group. Thus, the only significant result in the lagged analysis suggested a direction from affectionate touch to intimacy, albeit the effect was small: Bereaved women's higher-than-usual affectionate touch on one day was positively related to their intimacy on the next day.

**Table 5:** Multilevel model results for lagged effects between affectionate touch and intimacy.

	Loss Group			Comparison Group		
	*b*	*p*	95% CI	*b*	*p*	95% CI
**Intimacy_t_ => Affectionate Touch_t+1_**						
Actor effect (W)	−.05	.514	[−.18 - .09]	−.04	.472	[−.15 - .07]
Actor effect (M)	.09	.104	[−0.02 - .20]	.12	.069	[−.01 - .24]
Partner effect (W)	−.08	.166	[−.18 - .03]	.04	.387	[−.06 - .14]
Partner effect (M)	−.02	.769	[−.17 - .13]	.09	.195	[−.05 - .24]
**Affectionate Touch_t_ => Intimacy_t+1_**						
Actor effect (W)	**.10**	**.005**	**[.03 - .17]**	.04	.301	[−.04 - .13]
Actor effect (M)	−.02	.646	[−.09 - .06]	.01	.811	[−.07 – .09]
Partner effect (W)	.04	.216	[−.03 - .11]	−0.02	.529	[−.08 - .04]
Partner effect (M)	−.04	.439	[−.13 - .06]	.02	.700	[−.06 - .09]

*Note. W* = Women, *M* = Men. The bold result is significant. All reported results represent the lagged associations between affectionate touch and intimacy at the within-person level. Days 1–6 in the diary study are represented by “t,” whereas the consecutive days (Days 2–7) are represented by *t* + 1. We controlled for the autoregressive effects in the analysis.

Given that the majority of our sample experienced pregnancy loss (70%), we also conducted multigroup analyses to compare the results across pregnancy loss and during/after labor loss groups. Hence, we included parents who experienced pregnancy loss and during/after loss in the same model but constrained the actor and partner effects to be equal between and within levels across these two groups. The constrained and unconstrained models were significantly different from each other (Δχ²(25, *N* = 483)=277.26, *p* < .001), and hence we included both groups within the same multigroup model and estimated the parameters freely across each group. We present the results in the Supplemental Materials. While the women's partner effects at the between- and within-person levels were non-significant for pregnancy loss, these effects were positive and significant for couples who experienced child loss during/after labor. In addition, there was a significant partner effect of men's touch at the within-level for the pregnancy loss group. In contrast, this effect was non-significant for the during/after labor group.

Moreover, because the variances of some of the random slopes were significant in our original model for RQ2, we conducted separate multigroup analyses to test the moderating roles of loss-related characteristics (i.e., child's age, time since the loss, and experience of one vs. multiple child losses) in our results. Hence, we first created separate groups based on the frequency distribution of child's age and time since loss. For time since loss, 0–3.50 years (33.3%) were categorized into a group, and this was followed by 3.50 to 13.25 years (%33.4) and from 13.25 years to 51 years (%33.3) groups. Regarding the loss of a child during/after the loss, the child ages between 0 and 6 months (%50.4) were categorized into one group, and 6 months to 25 years (%49.4) were categorized into another group. We present the results in the Supplemental Materials. In these models, except for the non-significant actor effect of men whose child's age varied between 6 months and 25 years, all of the actor effects were significant. However, there were some differences in partner effects across different groups of child's age and time since loss.

## Discussion

In this paper, we investigated the association between affectionate touch and intimacy in bereaved (i.e., Loss Group) and non-bereaved couples (i.e., Comparison Group) using a 7-day dyadic diary study. We posed three research questions. First, we examined whether average affectionate touch levels differed across bereaved and non-bereaved couples, hypothesizing that bereaved couples would have lower affectionate touch levels than non-bereaved couples. Our results revealed that bereaved women, but not bereaved men, had lower levels of affectionate touch than their non-bereaved counterparts. Second, we investigated whether daily affectionate touch was positively associated with intimacy in both Loss and Comparison Groups. In line with our expectations, we found positive associations between daily affectionate touch and the provider's (i.e., actor effect) and receiver's (i.e., partner effect) intimacy, except for the non-significant partner effect of men in the Loss Group. Lastly, we explored whether partners' similarity in affectionate touch was associated with their intimacy levels and found that it is not the similarity at any level but both partners’ high levels of affectionate touch mattered for intimacy.

While there was a significant difference between bereaved and non-bereaved women's average affectionate touch levels, such difference did not emerge for men across the groups. However, the lower average touch in bereaved vs. non-bereaved women still occurred (albeit marginally) even when relationship length was controlled for. Moreover, descriptive examinations showed that bereaved men had higher levels of affectionate touch than bereaved women in both Loss and Comparison Groups. Although there are some mixed findings regarding the grief reactions of men and women, especially in response to non-child deaths, studies have highlighted that women may be more vulnerable to experiencing depression and post-traumatic symptoms than men after child loss (e.g., Pohlkamp et al., 2019). Such symptoms were shown to be negatively associated with parents' romantic relationship functioning (Lambert et al., 2012) and feelings of intimacy (Riggs, 2014). Bereaved women may experience their grief in a way that interferes with their willingness to provide affectionate touch towards their partner. Because bereaved mothers are more likely to avoid physical intimacy than bereaved fathers (Dyregrov & Gjestad, 2011), they may refrain from engaging in affectionate touch toward their partner.

The lack of difference in men's affectionate touch across bereaved and non-bereaved couples may be related to gender roles. For example, in a qualitative study conducted with Turkish parents who experienced pregnancy loss, bereaved men stated that they should not express their grief to fulfill the social expectations that fathers should be the ones staying strong and supporting their partners (Tanacıoğlu-Aydın & Erdur-Baker, 2022). Thus, bereaved men may hide their grief for their partner's sake and continue to engage in affectionate touch at a similar level to their non-bereaved counterparts to fulfill their societal roles.

In line with our second hypothesis, we found that affectionate touch benefits both bereaved and non-bereaved couples’ intimacy. Previous studies conducted with non-distressed couples highlighted the role of affectionate touch in enhancing feelings of support (Robinson et al., 2015) and security (Jakubiak & Feeney, 2016). In response to child loss, where the attachment behavioral system (see Bowlby, 1982/1969; Mikulincer & Shaver, 2009; Sbarra & Hazan, 2008) of the bereaved parents can become activated due to the complete separation from their (unborn) child, bereaved parents may engage in affectionate touch and benefit from its securing role to soothe the elevated stress. For instance, a recent study found that individuals who experienced different types of bereavement are likely to perceive a gentle touch and stroking of arms as consoling behaviors (Enmalm & Boehme, 2024). Furthermore, Jakubiak and Feeney (2017) proposed that affectionate touch can enhance the receivers' feelings of security, one of the mechanisms related to promoting closeness in romantic relationships. Thus, by affectionately touching their partner, bereaved parents might activate the virtuous cycle between intimacy and touch (Jolink et al., 2021).

Surprisingly, we found a non-significant partner effect of men's affectionate touch on women's intimacy in the Loss Group. In response to child loss, bereaved women may feel more guilt and responsibility than bereaved men (Li et al., 2014), perhaps especially in the case of pregnancy loss (Gold et al., 2018), which may negatively influence their willingness to be touched by their partner. We also do not know the participants’ interpretation of their partner's affectionate touch. For example, women may perceive their partner's behavior as a sign of detachment from their child and thus feel uncomfortable with this perceived unresponsive behavior (cf. Jolink et al., 2021). Alternatively, they may accept their partner's support provision through affectionate touch as given, considering that they are mostly evaluated as the “chief mourners,” especially in contexts with traditional gender roles (Tanacıoğlu-Aydın & Erdur-Baker, 2022). Such perceptions could explain the non-significant association between men's affectionate touch and women's intimacy.

We also found that, rather than the similarity of affectionate touch across partners at any level (i.e., low/medium/high levels), both partners’ engagement in higher vs. lower affectionate touch was linked to higher intimacy. Affectionate touch is positively associated with intimacy (Debrot et al., 2013) and relationship satisfaction (Carmichael et al., 2021; Floyd et al., 2009), and the benefits of affectionate touch on well-being are stronger among people who have highly satisfying relationships (Jakubiak & Feeney, 2021). Therefore, both partners' high engagement in affectionate touch may indicate a satisfactory romantic relationship where the partners can buffer each other's stress.

We also found that the actor effects are crucial to bereaved and non-bereaved couples' intimacy. Partner effects were weaker or non-significant. Although previous studies primarily focused on the impact of affectionate touch on the receiver, affectionate touch is also suggested to have similarly positive effects on the provider (Generous & Floyd, 2014). For example, Durbin et al. (2021) have found that enacting an affectionate touch toward the romantic partner is associated with the provider's feelings of intimacy. In addition, Debrot et al. (2013) found that affectionate touch was more strongly associated with the intimacy of the providers rather than the receivers. Bereaved parents may also engage in affectionate touch behaviors to elicit support from or to feel comforted by their partner (Forest et al., 2021; Jakubiak et al., 2021). Previous studies conducted on social support have also revealed that the benefits of support provision, such as experiencing higher levels of positive affect and lower rates of morality, are still observed in the case of a lack of reciprocal support (Brown et al., 2003; Knoll et al., 2007). Therefore, affectionate touch, due to its supportive nature, may play a similar role in bereaved couples’ relationships, and the providers may experience the positive consequences of affectionate touch regardless of their partner's affectionate touch level. Whether a mismatch across partners in affectionate could become detrimental to relationship quality, in the long run, is a question that awaits examination.

Although our bereaved sample predominantly consisted of parents who experienced pregnancy loss, our subsequent analyses on the comparison between parents who experienced pregnancy loss and during/after-labor loss highlighted the vital role of actor effects in both types of child losses, while the partner effects were inconsistent. In line with this finding, previous studies have indicated that pregnancy loss and during/after labor loss have similar psychological and relational consequences for bereaved parents (Gold et al., 2010; Murphy et al., 2014). The inconsistencies in partner effect may signal a more complex relationship between affectionate touch and intimacy for the receiver and may not depend solely on whether it is a pregnancy or during/after labor loss but also on other loss-related factors. Nevertheless, the benefits of affectionate touch, regardless of when the loss happened, can play an essential role in bereaved parents’ intimacy, especially for the provider.

Our results also showed that the strength of the positive association between one's affectionate touch and intimacy varied across bereaved parents, indicating heterogeneity and possible roles of other moderating factors. This finding is in line with previous studies, which found that not all bereaved parents have identical experiences or negative mental health consequences after child loss; instead, there are different groups of bereaved people regarding child loss's consequences (e.g., Infurna & Luthar, 2017). Because bereaved parents’ grief reactions are heterogeneous, how child loss unfolds in bereaved parents' romantic relationships can also be unique rather than a universal experience, as is also reflected in our results. However, despite some differences in partner effects, our results revealed consistently significant actor effects even when loss-related characteristics (i.e., experience of multiple child losses compared to a single loss, child's age, time passed since the loss) were used as a moderating variable in additional analyses.

The lagged effects between affectionate touch and intimacy showed that the association of bereaved women's daily affectionate touch was not limited to concurrent intimacy but extended to their intimacy on the next day, potentially signaling a directional influence of affectionate touch to intimacy rather than vice versa. This finding, combined with the result that affectionate touch behaviors occurred less often in bereaved women, suggests that bereaved women could be more selective about when they touch, but those selected physically close moments likely provide them with a prolonged emotional intimacy benefit. This speculation, however, awaits further experimental examinations.

### Strengths, limitations, and future directions

Our study has several strengths. First, our research adds to the literature by investigating bereaved parents' daily lives and romantic relationships using a dyadic diary design for the first time. This methodology allows a greater ecological validity and diminishes self-report bias (e.g., Reis et al., 2014). Second, we collected data from Turkish couples, an underrepresented context, contributing to the cultural diversity of bereavement and touch research. Lastly, because we collected data from both bereaved and non-bereaved couples, we were able to test the similarities and differences across groups. We found that bereaved couples were more similar than different from non-bereaved couples.

Nevertheless, our study has some limitations. First, because of the sample characteristics, such as the predominance of labor loss and a considerably long time after the loss, the results may not fully represent the daily experiences of recently bereaved parents after child loss. However, we should note that the wide range of time since loss is a common problem in bereavement research. For instance, to our knowledge, in the only experience sampling study (i.e., several assessments in a day) that examined prolonged grief symptoms in daily life, the time since the loss varied between 3 months and 46 years (Lenferink et al., 2022). Second, our sample mostly consisted of couples who had satisfying relationships, as reflected in the considerably high mean scores of relationship quality indicators, such as relationship satisfaction. Therefore, future studies could replicate these findings with bereaved couples who experience difficulties in maintaining their relationships, such as couples who seek family therapy. Third, despite the daily diary method, our study was correlational. For example, our finding about the effect of women's affectionate touch on the next day's intimacy should be tested with experimental designs.

There were also some differences in the sample characteristics between the Loss and the Comparison Groups (e.g., partners’ age, marriage duration), but controlling for these differential characteristics in the analyses did not change our findings. Due to practical issues stemming from the research design (dyadic diary study about a sensitive topic) and grant-related limitations (grant duration), we needed to start collecting data from the Comparison Group before data collection from the Loss Group ended. Future studies with more resources should aim to organize two consecutive data collection periods and target people with similar demographic characteristics. Moreover, our study focused only on the provision of affectionate touch. Future studies should also compare affectionate touch provision and receipt and how they differentially influence relationship dynamics in bereaved parents. In addition, future studies should investigate whether the discrepancies between the receiver's desire for affectionate touch and the partner's provision of affectionate touch influence couples’ relational outcomes. Moreover, whether the provision of one's affectionate touch can be accurately captured by their partner remains unknown. Therefore, future investigation of biases and accuracy in touch detection is awaited. Additionally, we used single-item measures to assess affectionate touch and intimacy. Although further investigation with more items is needed, single-item measures of romantic relationship quality have good psychometric qualities (Niehuis et al., 2024) and diminish participants’ burden in daily diaries (Bolger et al., 2003). Considering the sample characteristics and the topic's sensitivity, we hesitated to send the participants long surveys every day.

To conclude, we found that the known association between affectionate touch and intimacy in the general population extends to bereaved couples. In addition, we demonstrated that both partners’ high levels of affectionate touch contribute to their intimacy. Our findings highlighted the potential role of affectionate touch for future interventions in protecting and flourishing bereaved parents' romantic relationships after the loss of their child.

## Authors’ note

The authors disclose no conflict of interest. This research was funded by a grant from the Scientific and Technological Research Council of Turkey given to the second author (Grant number: 119K404). The syntax files and research materials are uploaded to the Open Science Framework repository (https://osf.io/m93vj/), but the dataset is not publicly shared because of its sensitivity.

## Declaration of competing interest

The authors declare that they have no known competing financial interests or personal relationships that could have appeared to influence the work reported in this paper.

## Appendix Supplementary materials

Image, application 1

## Footnotes

1 Cohen's d coefficients were calculated by subtracting the mean scores of compared variables of interests (e.g., women's vs. men's affectionate touch) and dividing it by the pooled standard deviation.
