# Supplementary material for: In contact with grief: Affectionate touch and intimacy in bereaved parents
Source: Int J Clin Health Psychol. 2024 Dec 12;24(4):100534. doi: 10.1016/j.ijchp.2024.100534 (PMC11699208; doi:10.1016/j.ijchp.2024.100534)
Supplement: Supplementary file 1 [file mmc1.docx]

**Supplemental Materials**

[Descriptive Statistics for Relationship Quality and Grief Variables 2](#_Toc183967961)

[Frequency Table for Religiosity and Socioeconomic Status Variables 3](#_Toc183967962)

[Summary Table for the Data Cleaning Procedure 4](#_Toc183967963)

[Affectionate Touch Distribution (%) 5](#_Toc183967964)

[Multilevel Power Curve 6](#_Toc183967965)

[Summary of the Results for Multilevel Models (RQ2) 7](#_Toc183967966)

[Wald Chi-Square Difference Test Results for DRSA Constraints 8](#_Toc183967967)

[Multilevel Model Results for Affectionate Touch’s Effect on Intimacy in Pregnancy Loss and During/After-Labor Loss Groups 9](#_Toc183967968)

[Multilevel Model Results for Affectionate Touch’s Effect on Intimacy in Parents who Experienced Multiple Losses and Single Loss 10](#_Toc183967969)

[Multilevel Model Results for Affectionate Touch’s Effect on Intimacy in Parents Based on Child’s Age 11](#_Toc183967970)

[Multilevel Model Results for Affectionate Touch’s Effect on Intimacy in Different Time Since Loss Categories 12](#_Toc183967971)

# **Descriptive Statistics for Relationship Quality and Grief Variables**

|  | Loss Group | | | |  | Comparison Group | | | |
| --- | --- | --- | --- | --- | --- | --- | --- | --- | --- |
|  | *M* | *SD* | *Observed Range* | *Possible Range* |  | *M* | *SD* | *Observed Range* | *Possible Range* |
| Relationship Satisfaction (W) | 6.91 | 2.26 | 1-9 | 1-9 |  | 7.32 | 1.80 | 1-9 | 1-9 |
| Relationship Satisfaction (M) | 7.80 | 1.60 | 1-9 | 1-9 |  | 7.70 | 1.55 | 1-9 | 1-9 |
| Trust (W) | 5.59 | 1.44 | 1-7 | 1-7 |  | 5.80 | 1.18 | 2-7 | 1-9 |
| Trust (M) | 6.32 | 0.97 | 2.14-7 | 1-7 |  | 6.11 | 1.06 | 2-7 | 1-9 |
| Closeness (W) | 5.38 | 1.79 | 1-7 | 1-7 |  | 5.42 | 1.58 | 1-7 | 1-9 |
| Closeness (M) | 5.99 | 1.26 | 1-7 | 1-7 |  | 5.63 | 1.54 | 1-7 | 1-9 |
| Grief Symptoms – TIG (W) | 3.20 | 1.25 | 1-5 | 1-5 |  | - | - | - | - |
| Grief Symptoms - TIG (W) | 2.76 | 1.37 | 1-5 | 1-5 |  | - | - | - | - |
| Grief Symptoms – TGI-SR (W)  Grief Symptoms – TGI-SR (M) | 2.42  1.93 | 1.01  0.85 | 1-4.72  1-4.56 | 1-5  1-5 |  | -  - | -  - | -  - | -  - |

*Note.* W=Women, M=Men, TIG=Texas Inventory of Grief (Faschingbauer et al., 1977), TGI-SR= Traumatic Grief Inventory Self-Report (Boelen et al., 2017). All of the variables mentioned in the table were measured during the cross-sectional survey.

Boelen, P. A., & Smid, G. E. (2017). The traumatic grief inventory self-report version (TGI-SR): Introduction and preliminary psychometric evaluation. *Journal of Loss and Trauma*, *22*(3), 196-212.

Faschingbauer, T. R., Devaul, R. A., & Zisook, S. (1977). Development of the Texas Inventory of Grief. *The American Journal of Psychiatry*. *134*(6), 696-698.

# **Frequency Table for Religiosity and Socioeconomic Status Variables**

|  | Women - Loss Group  N(%) | Men - Loss Group  N(%) | Women – Comparison Group  N(%) | Men – Comparison Group  N(%) |
| --- | --- | --- | --- | --- |
| **Religiosity** |  |  |  |  |
| 1 – Not at all | 8 (3.2) | 11 (4.7) | 26 (9.9) | 36 (13.8) |
| 2 | 13 (5.2) | 21 (9.0) | 26 (9.9) | 21 (8.1) |
| 3 | 21 (8.4) | 15 (6.4) | 28 (10.6) | 22 (8.5) |
| 4 | 45 (18.1) | 27 (11.5) | 45 (17.1) | 38 (14.6) |
| 5 | 69 (27.7) | 54 (23.1) | 78 (29.7) | 59 (22.7) |
| 6 | 40 (16.1) | 47 (20.1) | 29 (11.0) | 39 (15.0) |
| 7 – Very much | 30 (12.0) | 19 (8.1) | 11 (4.2) | 13 (5.0) |
| **Socioeconomic status** |  |  |  |  |
| 1 | 8 (3.2) | 3 (1.3) | 1 (0.4) | 1 (0.4) |
| 2 | 8 (3.2) | 12 (5.1) | 5 (1.9) | 8 (3.1) |
| 3 | 19 (7.6) | 10 (4.3) | 8 (3.0) | 16 (6.2) |
| 4 | 27 (10.8) | 29 (12.4) | 29 (11.0) | 27 (10.4) |
| 5 | 46 (18.5) | 41 (17.5) | 52 (19.8) | 45 (17.3) |
| 6 | 48 (19.3) | 44 (18.8) | 64 (24.3) | 58 (22.3) |
| 7 | 32 (12.9) | 33 (14.1) | 56 (21.3) | 52 (20.0) |
| 8 | 32 (12.9) | 21 (9.0) | 22 (8.4) | 8 (6.2) |
| 9 | 2 (0.8) | 2 (0.9) | 6 (2.3) | 1 (0.4) |
| 10 | 1 (0.4) | 0 (0) | 0 (0) | 4 (1.5) |

*Note.* In the Comparison Group, some participants had missing data in the religiosity question (20 women (%7.6); 40 men (%17.1)) and in the socioeconomic status question (20 women, (%7.6); 32 men (%12.3)). There were also participants in the Loss Group who had missing data in religiosity (23 women (%9.2); 32 men (%12.3)), and socioeconomic status (26 women (%10.4); 39 men (%16.7)) questions.

# **Summary Table for the Data Cleaning Procedure**

|  | Loss Group | Comparison Group |
| --- | --- | --- |
| 1) Number of rows in the original data | 3485 | 3423 |
| 2) Preview responses | 3 | 0 |
| 3) Did not enter the participant code, meaning that they did not see the rest of the survey | 115 | 49 |
| 4) Unrelated participant codes | 11 | 11 |
| 5) Deleted couples (e.g., participants who discussed their answers with their partners) | 68 | 49 |
| 6) Code-gender mismatch (Women participants’ codes should end with letter F and men participants’ codes should end with the letter M) | 24 | 34 |
| 7) Deleted rows based on the notes we took during the data collection (e.g., we learned that a third person read the questions to the participants) | 71 | 117 |
| 8) Entries at unrelated times (i.e., before 7 pm or after midnight) | 86 | 0 |
| 9) Repeated entries on the same days (we kept the first entries in terms of the start date) | 135 | 25 |
| 10) Other issues (e.g., cross-check with the cross-sectional data, participants who gave unreliable responses in cross-sectional survey was deleted in diary data) | 143 | 10 |
| 11) Filled in the survey for more than 7 days | 26 | 8 |
| **Final number of rows in the data** | 2803 | 3120 |

# **Affectionate Touch Distribution (%)**

|  | *<-0.5z* | *-0.5z - 0.5z* | >0.5z |
| --- | --- | --- | --- |
| Loss Group | 31.7 | 55.1 | 13.2 |
| Comparison Group | 29.6 | 54.4 | 16.0 |
| *Note.* The percentages indicate the distribution of differences between men’s and women’s standardized affectionate touch scores across seven days. The first column represents the percentage of couples where men had at least 0.5 z-score higher mean affectionate touch levels than women. The percentage in the second column suggests the z-score difference between partners varies between -.05 to .05. Third column shows the percentage of couples in which women have at least 0.5 z-score higher levels of mean affectionate touch than men. This distribution suggests that partners have varying affectionate touch levels, which is a precondition for the Response Surface Analysis (Shanock et al., 2010).  Shanock, L.R., Baran, B.E., Gentry, W.A., Pattison, S.C., & Heggestad, E.D. (2010). Polynomial regression with response surface analysis: A powerful approach for examining moderation and overcoming limitations of difference scores. *Journal of Business and Psychology, 25*(4), 543-554. | | | |
|  |  |  |  |

# **Multilevel Power Curve**


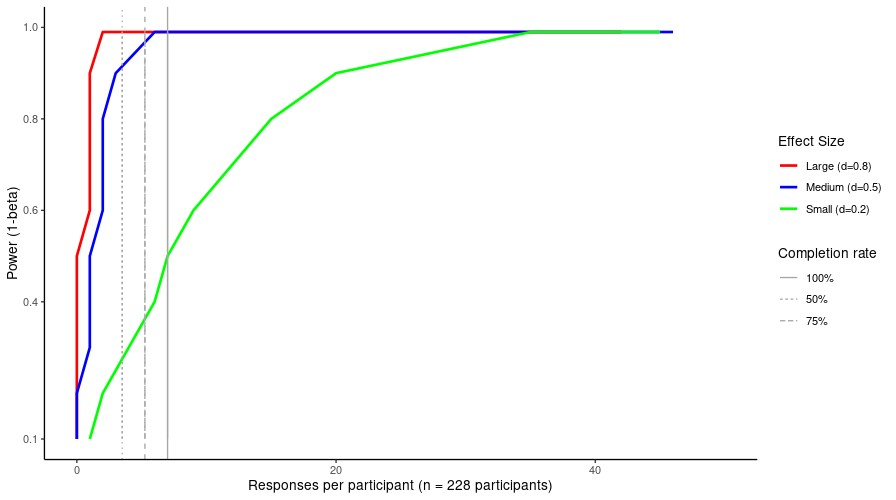


*Note.* This power curve for the multilevel model represents the sample’s power to detect small, medium, and large effect sizes. The figure was produced based on the sample characteristics of the men in the Loss Group to be conservative in terms of sample size (*N*=228, number of days=7, number of responses per day=1, ICC_intimacy_=.56).

<https://kleimanlab.org/resources/power-curves/>

# **Summary of the Results for Multilevel Models (RQ2)**

|  | Loss Group | Comparison Group | Pregnancy Loss Group | During/After Labor Group |
| --- | --- | --- | --- | --- |
| **Within-person level** |  |  |  |  |
| Actor effect | Positive for  both genders | Positive for  both genders | Positive for  both genders | Positive for  both genders |
| Partner Effect | Positive for  women | Positive for  both genders | Positive for  men | Positive for  women |
| **Between-person level** |  |  |  |  |
| Actor Effect | Positive for  both genders | Positive for  both genders | Positive for  both genders | Positive for  both genders |
| Partner Effect | Positive for  women | Non-significant | Non-significant | Positive for  women |

# **Wald Chi-Square Difference Test Results for DRSA Constraints**

|  | Loss Group | |  | Comparison Group | |
| --- | --- | --- | --- | --- | --- |
|  | Wald | p |  | Wald | p |
| Actor effects (b1_w_= b2_m_, b3_w_ = b5_m_) | 2.884 | 0.237 |  | 0.669 | 0.716 |
| Partner effects (b2_w_ = b1_m_, b5_w_ = b3_m_) | 1.624 | 0.444 |  | 0.500 | 0.779 |
| Interaction term (b4_w_ = b4_m_) | 0.000 | 0.994 |  | 0.143 | 0.705 |

*Note.* W = Women, M = Men. See Figure 1 in the main text for the regression coefficients b1-b5.

# **Multilevel Model Results for Affectionate Touch’s Effect on Intimacy in Pregnancy Loss and During/After-Labor Loss Groups**

|  | Pregnancy Loss | | |  | Labor/After Birth Loss | | |
| --- | --- | --- | --- | --- | --- | --- | --- |
|  | *b* | *p* | 95% CI |  | *b* | *p* | 95% CI |
| **Within-person level** | | | | | | | |
| Actor effect (W) | **.41** | **<.001** | **[.33 - .49]** |  | **.37** | **<.001** | **[.25 - .48]** |
| Actor effect (M) | **.38** | **<.001** | **[.30 - .46]** |  | **.43** | **<.001** | **[.31 - .55]** |
| Partner effect (W) | .04 | .104 | [-.01 - .09] |  | **.07** | **.045** | **[.00 - .15]** |
| Partner effect (M) | **.09** | **.049** | **[.00 - .17]** |  | -.03 | .529 | [-.11 - .05] |
| **Between-person level** | | | | | | | |
| Actor effect (W) | **.62** | **<.001** | **[.47 - .76]** |  | **.86** | **<.001** | **[.70 – 1.03]** |
| Actor effect (M) | **.49** | **<.001** | **[.35 - .60]** |  | **.41** | **.014** | **[.08 - .74]** |
| Partner effect (W) | .06 | .198 | [-.03 - .16] |  | **.20** | **.025** | **[.02 - .37]** |
| Partner effect (M) | .02 | .713 | [-.10 - .15] |  | -.18 | .115 | [-.41 – .04] |

*Note.* W=Women, M=Men. The bold results are significant.

# **Multilevel Model Results for Affectionate Touch’s Effect on Intimacy in Parents who Experienced Multiple Losses and Single Loss**

|  | Multiple Losses | | |  | Single Loss | | |
| --- | --- | --- | --- | --- | --- | --- | --- |
|  | *b* | *p* | 95% CI |  | *b* | *p* | 95% CI |
| **Within-person level** | | | | | | | |
| Actor effect (W) | **.42** | **<.001** | **[.31 - .53]** |  | **.39** | **<.001** | **[.31 - .47]** |
| Actor effect (M) | **.47** | **<.001** | **[.34 - .60]** |  | **.36** | **<.001** | **[.29 - .44]** |
| Partner effect (W) | .07 | .097 | [-.01 - .16] |  | .04 | .091 | [-.01 - .09] |
| Partner effect (M) | .06 | .384 | [-.07 - .19] |  | .06 | .085 | [-.01 - .14] |
| **Between-person level** | | | | | | | |
| Actor effect (W) | **.73** | **<.001** | **[.59 - .87]** |  | **.67** | **<.001** | **[.52 – .83]** |
| Actor effect (M) | **.54** | **<.001** | **[.33 - .76]** |  | **.45** | **<.001** | **[.29 - .61]** |
| Partner effect (W) | .08 | .144 | [-.03 - .18] |  | **.12** | **.031** | **[.01 - .22]** |
| Partner effect (M) | **.02** | **.014** | **[-.37 - -.04]** |  | .02 | .770 | [-.13 – .17] |

*Note.* W=Women, M=Men. The bold results are significant.

# **Multilevel Model Results for Affectionate Touch’s Effect on Intimacy in Parents Based on Child’s Age**

|  | Zero to 6 months | | |  | 6 months to 25 years | | |
| --- | --- | --- | --- | --- | --- | --- | --- |
|  | *b* | *p* | 95% CI |  | *b* | *p* | 95% CI |
| **Within-person level** | | | | | | | |
| Actor effect (W) | **.40** | **.012** | **[.09 - .71]** |  | **.31** | **<.001** | **[.14 - .49]** |
| Actor effect (M) | **.42** | **<.001** | **[.26 - .59]** |  | **.40** | **<.001** | **[.22 - .59]** |
| Partner effect (W) | **.14** | **.043** | **[.00 - .28]** |  | .01 | .822 | [-.09 - .12] |
| Partner effect (M) | -.05 | .745 | [-.34 - .24] |  | .03 | .713 | [-.11 - .16] |
| **Between-person level** | | | | | | | |
| Actor effect (W) | **.75** | **<.001** | **[.55 - .95]** |  | **.92** | **<.001** | **[.72 – 1.13]** |
| Actor effect (M) | **.57** | **<.001** | **[.29 - .85]** |  | .31 | .199 | [-.16 - .78] |
| Partner effect (W) | .06 | .275 | [-.05 - .16] |  | **.29** | **.028** | **[.03 - .54]** |
| Partner effect (M) | .01 | .945 | [-.34 - .36] |  | **-.31** | **.015** | **[-.33 – .36]** |

*Note.* W=Women, M=Men. The bold results are significant. The columns represent the child’s age. Based on the distribution of parents’ reports on their child’s age, we created two groups. While %50.6 of the bereaved parents experienced their loss when the child was aged between 0 to 6 months, the remaining %49.4 of the bereaved parents’ child’s age was between 6 months and 25 years.

# **Multilevel Model Results for Affectionate Touch’s Effect on Intimacy in Different Time Since Loss Categories**

|  | Within-person level | | |  | Between-person level | | |
| --- | --- | --- | --- | --- | --- | --- | --- |
|  | *b* | *p* | 95% CI |  | *b* | *p* | 95% CI |
| **Zero to 3.50 years** | | | | | | | |
| Actor effect (W) | **.52** | **<.001** | **[.39 - .64]** |  | **.67** | **<.001** | **[.49 - .84]** |
| Actor effect (M) | **.50** | **<.001** | **[.37 - .64]** |  | **.50** | **<.001** | **[.28 - .72]** |
| Partner effect (W) | .04 | .329 | [-.04 - .12] |  | **.14** | **.006** | **[.04 - .23]** |
| Partner effect (M) | .09 | .263 | [-.07 - .26] |  | .13 | .148 | [-.05 - .31] |
| **3.50 years to 13.25 years** | | | | | | | |
| Actor effect (W) | **.32** | **<.001** | **[.22 - .43]** |  | **.75** | **<.001** | **[.54 - .96]** |
| Actor effect (M) | **.34** | **<.001** | **[.22 - .46]** |  | **.52** | **<.001** | **[.35 - .69]** |
| Partner effect (W) | .04 | .138 | [-.01 - .08] |  | .06 | .406 | [-.09 - .21] |
| Partner effect (M) | .06 | .374 | [-.07 - .18] |  | -.15 | .061 | [-.30 - .01] |
| **13.25 years to 51 years** |  |  |  |  |  |  |  |
| Actor effect (W) | **.41** | **<.001** | **[.31 - .51]** |  | **.69** | **<.001** | **[.47 - .91]** |
| Actor effect (M) | **.38** | **<.001** | **[.29 - .47]** |  | **.44** | **.002** | **[.16 - .72]** |
| Partner effect (W) | .07 | .114 | [-.02 - .16] |  | .11 | .264 | [-.08 - .30] |
| Partner effect (M) | .00 | .971 | [-.11 - .11] |  | -.04 | .729 | [-.27 - .19] |

*Note.* W=Women, M=Men. The bold results are significant. The bolded cells in the first column represent the time since loss. Three groups were created based on the frequency distribution of the time since loss variable. First %33.3 of the cumulative distribution included losses from 0 and 3.50 years ago. The following %33.4 of the distribution included participants experiencing the loss between 3.50 and 13.25 years ago. Finally, the last %33.3 of the distribution consisted of participants whose time since loss varied between 13.26 years and 51 years.
